# *KRAS* Genotypic Changes of Circulating Tumor Cells during Treatment of Patients with Metastatic Colorectal Cancer

**DOI:** 10.1371/journal.pone.0104902

**Published:** 2014-08-19

**Authors:** Aristea Kalikaki, Helen Politaki, John Souglakos, Stella Apostolaki, Elisavet Papadimitraki, Nefeli Georgoulia, Maria Tzardi, Dimitris Mavroudis, Vassilis Georgoulias, Alexandra Voutsina

**Affiliations:** 1 Laboratory of Tumor Cell Biology, University of Crete, School of Medicine, Heraklion, Crete, Greece; 2 Department of Medical Oncology, University General Hospital of Heraklion, Heraklion, Crete, Greece; 3 Department of Pathology, University General Hospital of Heraklion, Heraklion, Crete, Greece; University of Patras, Greece

## Abstract

**Introduction:**

Circulating tumor cells (CTCs) could represent a non-invasive source of cancer cells used for longitudinal monitoring of the tumoral mutation status throughout the course of the disease. The aims of the present study were to investigate the detection of *KRAS* mutations in CTCs from patients with metastatic colorectal cancer (mCRC) and to compare their mutation status during treatment or disease progression with that of the corresponding primary tumors.

**Materials and Methods:**

Identification of the seven most common *KRAS* mutations on codons 12 and 13 was performed by Peptide Nucleic Acid (PNA)-based qPCR method. The sensitivity of the assay was determined after isolation of *KRAS* mutant cancer cells spiked into healthy donors' blood, using the CellSearch Epithelial Cell kit. Consistent detection of *KRAS* mutations was achieved in samples containing at least 10 tumor cells/7.5 ml of blood.

**Results:**

The clinical utility of the assay was assessed in 48 blood samples drawn from 31 patients with mCRC. All patients had *PIK3C*A and *BRAF* wild type primary tumors and 14 *KRAS* mutant tumors. CTCs were detected in 65% of specimens obtained from 74% of patients. *KRAS* mutation analysis in CTC-enriched specimens showed that 45% and 16.7% of patients with mutant and wild type primary tumors, respectively, had detectable mutations in their CTCs. Assessing *KRAS* mutations in serial blood samples revealed that individual patient's CTCs exhibited different mutational status of *KRAS* during treatment.

**Conclusions:**

The current findings support the rationale for using the CTCs as a dynamic source of tumor cells which, by re-evaluating their *KRAS* mutation status, could predict, perhaps more accurately, the response of mCRC patients to targeted therapy.

## Introduction

The association between *KRAS* mutations and response to EGFR inhibitors has been established in multiple studies; consequently, *KRAS* genotyping is recommended in all patients with metastatic colorectal cancer (mCRC) before any therapy that utilizes the EGFR-targeted monoclonal antibodies, cetuximab or panitumumab [1]. Nevertheless, not all patients with *KRAS* wild type tumors respond to EGFR-targeted therapies and the majority of the initially responsive patients experienced disease progression within 5 to 6 months [2].

Considering that most of the studies have been conducted using tissue obtained from the primary tumor whereas EGFR monoclonal antibodies have been used to treat the metastatic disease, it is possible that the lack of efficacy and/or the emergence of subsequent resistance may be due to genetic diversification of metastatic cells compared to their primary tumor counterparts or to dynamic variations in tumor genotype or phenotype that emerge during treatment.

Several studies have shown discordant mutation status between primary tumors and corresponding metastasis in a proportion (5%–30%) of CRC patients [3], [4], [5], [6]. Furthermore, recent studies suggest that acquired resistance is partly achieved by the selection of pre-existing minor sub-clones harboring mutations conferring resistance to anti-EGFR therapy [7], [8]. Because invasive biopsies of metastatic sites are not always feasible and cannot be easily performed repeatedly, circulating tumor cells (CTCs) in the peripheral blood of cancer patients, which are thought to mediate the hematogenous spread of disease to distant sites, may represent an alternative source of metastasizing tumor cells.

It is well documented that CTCs, as defined by the FDA-approved CellSearch System, could serve as a marker of micrometastatic tumor load associated with patients' prognosis and can accurately predict effectiveness of therapy in metastatic breast, colorectal, prostate and lung cancer [9], [10], [11], [12]. Previous studies in metastatic colorectal cancer suggested that the absolute number and the numerical variations of CTCs during disease progression or therapy can provide valuable information for the clinical outcome and the efficacy of administered treatments [13], [14], [15], [16], [17], [18]. However, CTCs cannot always be identified in metastatic patients, emphasizing the need to develop more sensitive and cancer type-specific CTC detection assays [19]. In this context, the identification of oncogenic mutations in CTCs could contribute to the improvement of existing detection methods. Moreover, genotyping of CTCs could possibly improve the monitoring of response to targeted therapies by identifying genomic profiles predictive of disease recurrence prior to clinical disease progression [20], [21], [22], [23], [24].

The aim of this study was to investigate the feasibility of detecting *KRAS* mutations in CTC-enriched fractions in patients with mCRC. Additional objectives were to evaluate whether *KRAS* mutation status of CTCs correlates with that of corresponding primary tumors and examine the genetic heterogeneity of CTCs in respect to *KRAS* mutation status during treatment.

## Materials and Methods

### Patients

Thirty-one patients with metastatic colorectal cancer were enrolled in the current study. In all patients, diagnosis was confirmed by histologic examination of the primary tumor before the initiation of any systemic therapy. All but one patient were treated with 5-FU-based first-line combination chemotherapy, with or without a biological agent (bevacizumab or panitumumab). Nineteen (55%) patients received an irinotecan-based combination and 11 (37%) an oxaliplatin-based regimen in the first-line setting (one patient did not receive any treatment). Additionally, 25 (83%) patients received bevacizumab and two (7%) panitumumab. At the time of analysis, 19 patients presented disease progression to first-line treatment and 12 of them were treated with a second-line chemotherapy regimen; five out of these 12 patients were treated with panitumumab in combination with chemotherapy.

Peripheral blood was analyzed for the presence of CTCs before initiation of first-line treatment in 12 patients, at the time of progression on first-line treatment in 9 and at any time during treatment in 18 patients.

All patients as well as 16 healthy blood donors, who had no known illness and no history of malignant disease, were tested for the presence of CTCs using the CellSearch Epithelial Cell Kit (Veridex LLC). Peripheral blood was obtained from mCRC patients (7.5 ml) and healthy donors (23 ml, for use in spiking experiments) by vein puncture in the specific CellSave tubes; in order to avoid contamination of the samples with epithelial cells the first 5 ml of the blood was discarded. All tests were performed within 72 hours from the blood draw according to the manufacturer's instructions. Cells which were CK(+)/CD45(−) and had a DAPI-positive intracellular nucleus were characterized as CTCs by experienced biologists (E.P. and S.A.).

Since this study was a pilot feasibility study, there was no a statistically sample size estimation and specimen collection was based on the availability of CellSearch cartridges.

### Ethics statement

All patients gave their written informed consent to participate in the study which has been approved by the Ethics and Scientific Committees of the University General Hospital of Heraklion, Crete, Greece; the manuscript was prepared according to the REMARK criteria [25].

### Cell lines

Cancer cell lines harboring *KRAS* mutations [LS174T, Human colon adenocarcinoma, c.35G>A (p.G12D); HCT116, Human colon adenocarcinoma, c.38G>A (p.G13D); HUP-T3, Human pancreatic adenocarcinoma, c.34G>C (p.G12R); KYSE410, Human oesophageal squamous cell carcinoma, c.34G>T (p.G12C); A549, Human alveolar adenocarcinoma, c.34G>A (p.G12S); SW403, Human colon adenocarcinoma, c.35G>T (p.G12V) and RPMI8226, Human myeloma, c.35G>C (p.G12A)] or wild type for *KRAS* (HT-29, Human colon adenocarcinoma)] originated from the American Type Culture Collection (ATCC, USA) and were kindly provided from Prof. A. Jung (Institute of Pathology, Ludwig-Maximilian-University, Munich, Germany). All cell lines were cultured in flasks according to supplier's recommendations, before subsequent harvesting using 0.25% trypsin and 5 mmol/L EDTA (GIBCO-BRL). Authentication was done by determining the *KRAS* mutational status of each cell line by Sanger sequencing. All cell lines except SW403 were heterozygous for *KRAS* mutations and revealed the expected genotype.

### DNA isolation from CTC-enriched fractions and tissues

Following CTC counting, the captured cells were transferred from the chamber to a 2 ml tube and subjected to a Proteinase K digestion at 65°C for 2–5 h; DNA isolation was performed using the Epicentre MasterPure Complete DNA & RNA Purification Kit (Epicentre Biotechnologies, Madison, WI, USA) according to the manufacturers' instructions and the extracted DNA was quantified using a NanoDrop ND1000 (NanoDrop Technologies, USA) and stored at −20°C until used. The average yield of DNA was ∼1.5 µg; however, DNA quantification by UV spectroscopy is not precise due to the detection of single stranded DNA, free nucleotides or RNA in the sample.

Formalin-fixed paraffin-embedded (FFPE) primary tumor-tissue was evaluated histologically by a pathologist (MT) and micro-dissection was performed to increase the percentage of tumor cells. Three 5 µm tissue sections were deparaffinised by xylene and ethanol washes and subjected to a Proteinase K digestion overnight at 56°C DNA was then purified using a QIAamp DNA Micro Kit (Qiagen, Germany).

### Mutation analysis in primary tumors

In consenting patients, primary tumors were evaluated for mutations in *KRAS*, *PIK3CA* and *BRAF* by both Sanger sequencing and methodologies with high sensitivity as previously described [26].

### *KRAS* mutation assay

A mutation assay that combined the peptide nucleic acid (PNA)-mediated PCR clamp with TaqMan-MGB allelic discrimination assays has been previously developed to detect the seven more common *KRAS* mutations [6].The thermal conditions for PNA mediated PCR clamping for each of the seven *KRAS* mutant templates were further optimized by using a PNA labeled with a fluorescent dye as both sensor probe and PCR clamp according to Luo JD et al [27]. The greatest selectivity, i.e. high Ct value in the wild-type probe and low Ct value in the mutant probe was achieved at 62°C with 200 nM PNA for the c.35G>A (p.G12D), c.38G>A (p.G13D) and c.35G>T (p.G12V) and 150 nM PNA for the c.34G>C (p.G12R), c.34G>T (p.G12C), c.34G>A (p.G12S) and c.35G>C (p.G12A) *KRAS* mutant templates respectively. The reactions were performed on Applied Biosystems 7900HT Real-Time PCR System in 384-well plates in a total volume of 5 µl, containing 2 µl (approximately 1/8 of total extracted DNA from CTC-enriched specimens and 20 ng extracted DNA from FFPEs) DNA, 2.5 µl 2X TaqMan genotyping master mix (Applied Biosystems, USA), 0.25 µl of genotyping assay mix and 150 or 200 nm PNA; in parallel, a non-PNA-clamp reaction was performed. The PCR conditions were 95°C for 10 min, followed by two-step cycling: 50 cycles of 92°C for 15 s, and 62°C for 90 s. All samples were run at least in duplicates. Ct values were obtained from the instrument's real-time PCR data collection software using 0.2 manual threshold. A positive control for each *KRAS* mutation (model samples that comprised mixtures of cell lines with mutated/wild type ratios 1/100 and 1/500) and a negative control (*KRAS* wild type cell line) in total input of 50 ng were included in each run.

The ΔCt = Ct_mut probe (+PNA)_ - Ct_wt probe (-PNA)_ value was computed for each sample; Ct_mut probe (+PNA)_ and Ct_wt probe (-PNA)_ denoted the Ct (cycle threshold Ct, is termed as the cycle number at which a signal is detected above background fluorescence) values for the mutant and wt probes of the reactions with and without PNA, respectively. The Ct_mut probe (+PNA)_ reflects the amount of *KRAS* mutant DNA within the sample while the Ct_wt probe (-PNA)_ reflects the amount of amplifiable template derived from the varying numbers of contaminating leucocytes in CTC fraction [28].

### Assay validity

**Figure 1:**
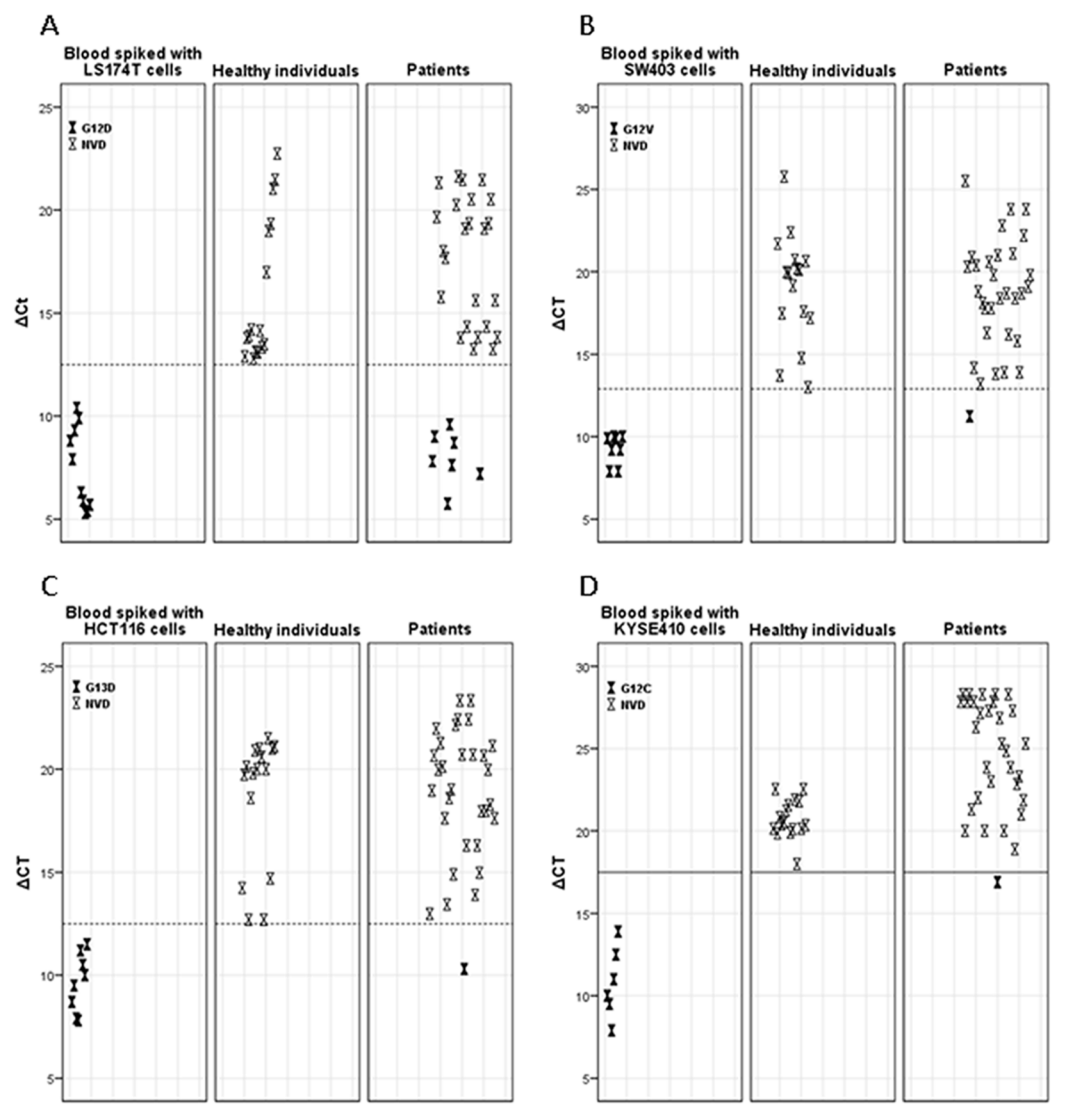
Representative plots showing the *KRAS* mutation status of CTC-enriched specimens. Graphs showed the ΔCt values (*y* axis) versus the CTC-enriched specimens (*x* axis) of mCRC patients (n = 31), healthy individuals (n = 16) and model samples spiked with 100 and 10 cells from cell lines (LS174T, SW403, KYSE410 and HCT116) harboring hotspot *KRAS* mutations c.35G>A; p.G12D (A), c.35G>T; p.G12V (B), c.38G>A; p.G13D (C) and c.34G>T; p.G12C (D). Cutoff threshold is represented by dot-line; specimens with ΔCt below the threshold are considered mutant; ΔCt = Ct_mut probe (+PNA)_ – Ct_wt probe (-PNA)_; NVD, no variant detected.

Validity of the assay result for each sample was determined by the Ct_wt probe (-PNA)_ value that should be 24≤Ct_wt probe (-PNA)_ <32; if the Ct_wt probe (-PNA)_ in a sample is greater than 32, the assay result is not reliable because of a low amount of DNA or failed target amplification. Efficient PCR PNA clamping was confirmed by a Ct_wtprobe (+PNA)_ value >45. The *KRAS* mutation status was determined by comparison of the ΔCt values to previously defined ΔCt cutoff values for each of the seven more common *KRAS* mutations. The cutoff values for each mutation assay have been determined from analysis of 16 healthy donors' blood samples following CellSearch analysis. As cutoff value was defined the ΔCt corresponding to the maximum specificity; that is, no mutation detected in 100% (16/16) of healthy donors (Table 1, Figure 1).

**Table 1:** Analytical sensitivity of *KRAS* mutation detection assay.

	*KRAS* mutation detection assay						
% of mutant/wild type DNA	G12D	G13D	G12R	G12C	G12S	G12V	G12A
**100**	−1.0	0.3	0.3	1.1	−1.8	0.6	−1.4
**50**	3.5	3.9	3.9	4.2	2.5	4.9	3.9
**10**	4.4	6.7	5.9	6.6	4.1	6.1	5.7
**2**	7.8	9.9	7.8	9.0	6.0	9.3	8.2
**1**	8.6	10.6	9.6	10.6	7.2	10.4	9.4
**0.5**	9.9	11.2	10.7	11.7	8.8	11.5	10.8
**0.1**	**11.9**	14.3	**13.8**	**15.5**	**12.5**	14.1	**13.4**
**0**	13.8	20.9	25.4	24.8	15.8	24.8	25.0
**Cut off**	**12.5**	**12.5**	**19.0**	**17.5**	**13.7**	**12.9**	**18.0**

For every assay, 0.1% to 100% of mutant cell line gDNA was mixed into a background wild type gDNA keeping the total input constant at 20 ng. The numbers indicate the ΔCt values; the cutoff value (mutated threshold) for each assay is also shown.

### Analytical specificity and sensitivity

The analytical specificity of the seven assays was individually evaluated using genomic DNA (gDNA) isolated from *KRAS* wild-type cell line (HT29) or positive for specific *KRAS* mutations; the lower limit of detection for all assays was 0.015 ng. To assess analytical sensitivity, each of the seven *KRAS* mutant cell line gDNA was serially diluted over a range of three different concentrations (100 ng, 50 ng and 20 ng) of wild-type gDNA provided by the HT-29 cell line to give mutation/wild-type ratios of 100%, 50%, 10%, 2%, 1%, 0.5%, 0.1% and 0%. All assays have a sensitivity of 0.1%, keeping the total input constant at 20 ng, except the assays for the detection of p.G13D and p.G12V that have a sensitivity of 0.5% (Table 1).

### Cross reactivity

Cross-reactivity tests were performed for all mutation assays that potentially identify another assay's template due to imperfect base-pairing and read-through. The results indicate that the G12D assay shows significant cross-reactivity with the G12V and G12A mutant templates, the G13D assay with the G12S mutant template, the G12R assay with the G12C and G12S mutant templates and the G12C assay with the G12S mutant template (Table 2).

**Table 2:** Cross reactivity pattern of *KRAS* mutation detection assay.

		*KRAS* mutation detection assay						
		G12D	G13D	G12R	G12C	G12S	G12V	G12A
2**0 ng DNA input**	**LS174T (c.35G>A; p.G12D)**	−1.6	—	—	—	—	—	—
	**HCT116 (c.38G>A; p.G13D)**	—	-1.7	—	—	—	—	—
	**HUPT3 (c.34G>C; p.G12R)**	—	—	-0.5	14	—	—	—
	**KYSE410 (c.34G>T; p.G12C)**	—	—	5.8	0.9	—	—	—
	**A549 (c.34G>A; p.G12S)**	—	6.4	9.3	5.5	−2.9	—	—
	**SW403 (c.35G>T; p.G12V)**	4.5	—	—	—	—	0.6	—
	**RPMI8226 (c.35G>C; p.G12A)**	0.5	—	—	—	—	—	−18.1

### Intra- and inter-assay precision

Intra-assay precision has been evaluated by analyzing model samples run in triplicate in the same experiment using serial dilution (100%, 50%, 10%, 2%, 1%, 0.5% and 0.1%) of *KRAS* mutant DNAs, as mentioned above, in 20 ng of background *KRAS* wild type DNA. The ΔCt coefficients of variation for the *KRAS* mutated DNA ranged between 0.3% and 2.5%. Inter-assay reproducibility has been similarly measured by calculating the ΔCt coefficients of variation of triplicate samples run in different days using the same serial dilutions of mutated DNA. The ΔCt coefficients of variation for the *KRAS* mutated DNA ranged between 0.4% and 2.5%. Furthermore, in model samples with a mutated/wild type ratio 0.5%, the overall mutation assay failure rate was 0%.

## Results

### Mutation assay optimization

The sensitivity of our experimental approach to determine accurately the *KRAS* mutation status in a limited number of CTCs present among normal blood cells in circulating blood was validated by spiking *KRAS* mutant cancer cells into the blood of healthy donors. Approximately 100 and 10 tumor cells were spiked into 7.5 ml of blood in CellSave tubes and analyzed by CellSearch within 2 days, in at least 3 independent experiments. The average percent recovery for the 100 and 10 spiked cells [LS174T, c.35G>A (p.G12D), n = 5 experiments; HCT116, c.38G>A (p.G13D), n = 4 experiments; SW403, c.35G>T (p.G12V), n = 4 experiments and KYSE410, c.34G>T (p.G12C), n = 3 experiments] was 95% (range, 70%–100%) and 92.5% (range, 25%–130%), respectively. The broad range of recovery can be attributed to the error associated with spike-in low numbers of cells. Following CTC enumeration by the CellSearch Epithelial Cell kit, DNA was extracted from the captured cells and specimens were analyzed for all seven *KRAS* mutations. By this assay, *KRAS* mutations could be consistently identified from 10 spiked tumor cells.

### Patients' characteristics and evaluation of CTCs

Patient's demographics, clinical and pathological characteristics are listed in Table 3. *KRAS* mutations were identified in the primary tumors of 45% of patients; eight (57%) patients' primary tumor harbored the c.35G>A (p.G12D) mutation, three (21%) the c.35G>T (p.G12V), one (7%) the c.38G>A (p.G13D), one (7%) the c.34G>T (p.G12C) and one (7%) the c.35G>C (p.G12A). All tumors were *PIK3CA* and *BRAF* wild type. The median time between surgical resection of the primary tumor and analysis of CTCs was 6 months (range 1 to 134).

**Table 3:** Pathological and clinical characteristics of mCRC patients.

Characteristics	No. of patients	%
**Age** (years)		
Median (range)	66 (28–85)	
**Gender**		
Female	9	29.0
Male	22	71.0
**Primary tumor site**		
Right colon	12	38.7
Left colon	18	58.1
Unknown	1	3.2
**Tumor**		
T2	2	6.5
T3	18	58.1
T4	3	9.7
Tx	8	25.8
**Nodal**		
N0	5	16.1
N1	4	12.9
N2	12	38.7
N3	1	3.2
Nx	9	29.0
**Lymphovascular invasion**		
Yes	15	48.4
No	16	51.6
**Tumor grade**		
Low/Moderate	20	64.5
High	11	35.5
**Adjuvant therapy**		
Yes	5	16.1
No	26	83.9
**1^st^ line regimens**		
Folfiri plus Bevacizumab	17	56.7
Folfox plus Bevacizumab	9	30.0
Folfiri plus Panitumumab	1	3.4
Folfox plus Panitumumab	1	3.4
Folfiri	1	3.4
Folfox	1	3.4

A total of 48 blood samples were obtained from 31 patients for CTC enumeration using CellSearch (Table 4). Twelve (39%) patients were chemotherapy naïve at the time of first blood draw. Twelve (71%) and 11 (79%) patients with *KRAS* wild type and mutant primary tumors, respectively, had detectable CTCs; a median of 9 (range 2 to 660) and 7 (range 1 to 865) CTCs were detected in patients with *KRAS* wild type and mutant primary tumors, respectively. In total, 1 or more CTCs could be detected in 31 (65%) blood samples obtained from 23 (74%) patients (Table 4). *KRAS* mutation analysis on codons 12 and 13 was performed in all specimens with detectable CTCs (Figure 1).

**Table 4:** *KRAS* mutation status in primary tumor and corresponding CTC-enriched samples in mCRC patients.

	Primary tumor	CTC-enriched fraction		
Patient ID	*KRAS* status	CTC count a	*KRAS* status	Time point
1	c.35G>A; p.G12D	102	c.35G>A; p.G12D	Prior 1^st^ line Folfox/Bevacizumab
		12	c.35G>A; p.G12D	Post cycle 1 of 1^st^ line
		9	NVD	Post cycle 3 of 1^st^ line
		42	c.35G>A; p.G12D	Post cycle 8 of 1^st^ line
2	c.35G>A; p.G12D	243	NVD	Prior 1^st^ line Folfiri
		865	NVD	Progression on 1^st^ line therapy
3	c.35G>A; p.G12D	1	NVD	Prior 1^st^ line Folfiri/Bevacizumab
		4	NVD	Post cycle 3 of 1^st^ line
4	c.34G>T; p.G12C	2	NVD	Prior 1^st^ line Folfox
5	c.35G>A; p.G12D	3	c.35G>A; p.G12D	Prior 1^st^ line Folfiri/Bevacizumab
6	c.35G>A; p.G12D	11	c.35G>A; p.G12D	Post cycle 3 of 1^st^ line Folfox/Bevacizumab
7	c.35G>C; p.G12A	2	NVD	Post cycle 3 of 1^st^ line Folfox/Bevacizumab
		0	-	Progression on 1^st^ line therapy
		2	NVD	Post cycle 3 of 2^nd^ line Folfiri/Bevacizumab
8	c.35G>T; p.G12V	7	c.35G>T; p.G12V	Progression on 1^st^ line Folfiri/Bevacizumab
		0	-	Post cycle 3 of 2^nd^ line Folfox/Bevacizumab
9	c.35G>A; p.G12D	0	-	Post cycle 8 of 1^st^ line Folfiri/Bevacizumab
		0	-	Progression on 1^st^ line therapy
10	c.38G>A; p.G13D	9	c.38G>A; p.G13D	Post cycle 6 of 1^st^ line Folfiri/Bevacizumab
11	c.35G>A; p.G12D	0	-	Prior 1^st^ line Folfiri/Bevacizumab
12	c.35G>A; p.G12D	5	NVD	Prior 1^st^ line Folfiri/Bevacizumab
13	c.35G>T; p.G12V	4	NVD	Post cycle 1 of 1^st^ line Folfiri/Bevacizumab
		0	-	Post cycle 3 of 1^st^ line Folfiri/Bevacizumab
14	c.35G>T; p.G12V	0	-	Post cycle 6 of 1^st^ line Folfiri/Bevacizumab
		0	-	Progression on 1^st^ line therapy
		0	-	Palliative care
15	wt	3	NVD	Maintenance therapy with Panitumumab
		42	c.35G>A; p.G12D	Progression on Panitumumab
		10	c.35G>A; p.G12D	Post cycle 2 of 2^nd^ line Folfiri/Panitumumab
16	wt	7	c.34G>T; p.G12C	Post cycle 1 of 1^st^ line Folfiri/Bevacizumab
17	wt	0	-	Post cycle 3 of 1^st^ line Folfiri/Bevacizumab
18	wt	10	NVD	Without treatment
19	wt	660	NVD	Prior 1^st^ line Folfox/Bevacizumab
20	wt	3	NVD	Prior 1^st^ line Folfox/Bevacizumab
21	wt	0	-	Prior 1^st^ line Folfiri/Bevacizumab
		2	NVD	Post cycle 3 of 1^st^ line therapy
22	wt	0	-	Prior 1^st^ line Folfiri/Bevacizumab
		0	-	Post cycle 4 of 1^st^ line therapy
23	wt	0	-	Prior 1^st^ line Folfox/Bevacizumab
24	wt	17	NVD	Post cycle 6 of 1^st^ line Folfiri/Bevacizumab
		0	-	Follow up
25	wt	173	NVD	Post cycle 2 of 1^st^ line Folfiri/Bevacizumab
26	wt	11	NVD	Post cycle 6 of 1^st^ line Folfox/Bevacizumab
27	wt	8	NVD	Post cycle 6 of 1^st^ line Xeloda/Bevacizumab
28	wt	0	-	Progression on 1^st^ line Folfiri/Bevacizumab
29	wt	4	NVD	Progression on 1^st^ line Folfox/Panitumumab
30	wt	3	NVD	Progression on 1^st^ line Folfiri/Panitumumab
31	wt	0	-	Progression on 1^st^ line Folfiri/Bevacizumab

a CTC count using CellSearch system depicted as number of cells per 7.5 mL whole blood. Abbreviations: wt; wild type, NVD; no variant detected.

### *KRAS* mutations in patients' CTC-enriched samples

*KRAS* mutations could be identified in the CTCs of five (45%) patients whose primary tumors had mutated *KRAS*. In particular, before the initiation of 1^st^ line chemotherapy only two of the six chemotherapy naïve patients (#1 and #5) harbored CTCs with detectable mutations while at the time of disease progression, mutations could be identified in one (#8) of the two patients; mutations were also detectable during the follow-up of 1^st^ line treatment in two patients (#6 and #10) (Table 4).

*KRAS* mutations could also be identified in the CTCs of two (17%) patients (#15 and #16) whose primary tumors had no detectable mutations; in both patients, CTCs were collected during the monitoring of 1st line chemotherapy (Table 4).

### *KRAS* mutational status of CTCs in serial blood samples

Four serial blood samples of patient #1 had detectable CTCs; *KRAS* mutations were documented even after the administration of the first chemotherapy cycle despite the important decrease of the number of CTCs; treatment continuation (after the 3^rd^ cycle) resulted in further decrease of the CTC number and undetectable *KRAS* mutations in CTC-enriched specimens while assessment of treatment response revealed a partial response; after the 8^th^ chemotherapy cycle, the number of CTCs was increased and *KRAS* mutations could be detected again while the patient still remained in partial response (Table 4).

In patient # 15 who had a *KRAS* wild type primary tumor, no *KRAS* mutations could be detected in the CTC-enriched specimen when the patient was in partial response and under treatment with maintenance panitumumab; however, after 4 months of treatment, at the time of disease progression, as documented by the radiological worsening of hepatic lesions and clinical appearance of ascites, the number of CTCs was increased and *KRAS* mutations could be identified in CTCs-enriched cell fraction. *KRAS* mutations were further detectable after the 2^nd^ cycle of salvage chemotherapy despite the decrease of the CTC number (Table 4).

## Discussion

Mutations in *KRAS* result in the constitutive activation of the RAS/MAPK pathway and predict lack of response to anti-EGFR monoclonal antibodies. To date, therapy decisions rely mainly on *KRAS* mutation status of the primary tumor; however, genetic changes occurring during disease progression and acquired resistance to treatment may alter tumor's biology. Therefore, an important question concerns the possibility to use CTCs as a non-invasive alternative of a new biopsy which could be more representative of the current biological status of tumor cells and might be used as a biomarker of either sensitivity or acquired resistance to EGFR targeted therapy.

The results of the current study clearly indicate the feasibility to determine the *KRAS* mutation status in CTC-enriched blood samples in the context of routine clinical practice, following CTCs enumeration by the CellSearch system. The presented data strongly support the hypothesis that CTCs may represent a real-time source of liquid biopsy which may allow the dynamic genotyping of tumor cells during treatment. The data also indicate that the established assay provides a detection sensitivity of approximately 10 mutated cells/7.5 ml of blood, without the need of whole genome amplification, and offers the possibility for a non-invasive, rapid and low-cost serial monitoring of the *KRAS* mutation status in codons 12 and 13 during the course of treatment.

*KRAS* mutations were identified even in clinical specimens containing as low as 3 CTCs/7.5 ml of blood; we cannot exclude that this may be due to the presence of *KRAS* mutations in CTC fragments which are not considered as real CTCs during the documentation and enumeration of CTCs by the CellSearch system and/or cell free DNA (cfDNA) adsorbed to the surface of contaminating leukocytes [29]. It is to note that prior studies have shown that both CTCs and CTC fragments correlate with patient outcomes in prostate cancer [30].

Despite the small sample size and the heterogeneous patient population analyzed, the present study provides evidence that the *KRAS* mutation status of CTCs may substantially differ from that of the corresponding primary tumor. CTCs with no detectable *KRAS* mutations were obtained from six out the 11 patients with mutant primary tumors; this could be explained by the intra-tumoral heterogeneity and/or the enrichment of minor preexisting clones with increased metastatic potential during disease progression. Nevertheless, in three of the six discordant cases, the resection and examination of the primary tumor for *KRAS* mutations was performed only one month before the analysis of CTCs (data not shown), indicating possibly a model of parallel evolution of the primary tumor and metastasis. Previous studies have shown that the mutation status of CTCs does not always reflect that of the corresponding metastasis [31]; therefore, the comparison of the *KRAS* mutation status of primary tumor, corresponding metastases and serial CTC-enriched blood specimens might shed light to the origin of metastases.

Although the conditions and the rate of genotype conversion are not well understood, the current study suggests that CTCs of different mutation status may arise during treatment in the same patient (patients #1 and #15). Indeed, it could be hypothesized that treatment, with or without targeted agents, by eliminating some chemotherapy-sensitive clones may allow the emergence of other low frequency clones that differ from the predominant tumoral cells in respect to the mutation status. This hypothesis is strongly suggested by the presence of *KRAS* mutations in CTCs of two out the 12 patients with *KRAS* wild type primary tumors who were treated with regimens incorporating panitumumab or bevacizumab (patients #15 and #16). Nevertheless, the failure of detecting mutations in the CTC-enriched samples could also be attributed to the low frequency of CTCs harboring mutations as well as to methodological limitations; the FDA approved CellSearch Epithelial Cell Kit, which is reported to be inferior to CellSearch Epithelial Cell Profile kit in terms of CTCs' molecular characterization [32]. However, it has been shown that CTCs captured with the CellSearch Epithelial Cell Kit can be successfully analyzed by next-generation sequencing methodologies [33]. Furthermore, a subpopulation of CTCs could not be detected by CellSearch due to insufficient expression of EpCAM and/or Cytokeratins; though, a recent study in SCLC demonstrated that CTCs, expressing EpCAM, captured with CellSearch system can form tumors in immunocompromised mice [34].

Previous studies have used different methodologies in order to isolate and molecularly characterize the CTCs. In metastatic breast and prostate cancer, genomic profiling of CTCs isolated by immunomagnetic enrichment and fluorescence activated cell sorting revealed new genomic changes occurring during disease progression [35], [36]. Using a microfluidic CTC capture device, the *TMPRSS2-ERG* fusion, the TKIs sensitizing *EGFR* activating mutations and the *EGFR* T790M TKI-resistance mutation were detected in CTCs from patients with prostate and lung cancer metastatic disease respectively, whereas mutations of the *AR*, *KRAS* and *BRAF* genes have been identified in CTC-enriched samples isolated from prostate and mCRC patients, respectively, using the CellSearch Profile Kit [24], [37], [38], [39]. Genotyping of single CTCs isolated by the CellSearch or the IsoFlux system in patients with mCRC confirmed an intra- and inter- patient heterogeneity based on the *PIK3CA* and *KRAS* mutation status [22], [31]; moreover, different genetic alterations on single CTCs have already been reported in patients with breast and esophageal cancer [23], [40].

Previous reports suggested a link between the presence of CTCs and the cell free DNA (cfDNA) in the serum or plasma of cancer patients [41]. *KRAS* mutations detected in the serum of patients with mCRC have been proposed to monitor response to treatment before the clinical appearance of disease progression whereas in NSCLC patients it has been reported a higher sensitivity for EGFR mutation detection in cfDNA than in CTCs [7], [8], [42]. Nevertheless, the origin of cfDNA is not well understood since it might be derived from apoptotic tumor cells, apoptotic leucocytes produced by the cytotoxic therapy as well as by exosomes or CTCs released from tumor cells in the bloodstream. Although the isolation and analysis of cfDNA is simpler and more reproducible than isolating and genotyping CTCs, the molecular characterization of CTCs in addition to being useful for monitoring treatment failure or disease relapse may also provide information to guide optimal treatment selection and/or development of novel therapies.

In conclusion, the presented data describe a simple methodology based on the daily use of the CellSearch platform for the identification of *KRAS* mutational status of CTCs from patients with metastatic colorectal cancer and provide a rationale for considering re-assessment of *KRAS* mutational status in CTCs in order to better predict response to anti-EGFR therapy.
